# Systems architecture for integrated care

**Published:** 2012-06-15

**Authors:** David Kwo

**Affiliations:** Managing Director, Ubiquity Services Ltd, Surrey, UK

**Keywords:** telehealth, telecare, systems architecture, integrated health care

## Abstract

**Introduction:**

Telehealth and telecare projects do not always pay enough attention to the wider information systems architecture required to deliver integrated care. They often focus on technologies to support specific diseases or social care problems which can result in information silos that impede integrated care of the patient. While these technologies can deliver discrete benefits, they could potentially generate unintended disbenefits in terms of creating data silos which may cause patient harm or at least impede the ability of the clinician, carer or even patient to treat the patient in an integrated fashion. For instance, if clinical data (vital signs, assessments, medications, allergies) are captured in a telehealth or telecare system, but not integrated with the patient record in the GP or hospital system (or vice versa), then drug or treatment contra-indications could be missed and the patient put at risk.

**Architectures:**

Telehealth and telecare technologies need to be designed and developed within information systems architectures that support the wider objectives of integrated care. Such architectures should be clear about the integration trade-offs implicit in the technology designs between: practical and earlier delivery of benefits in the short-term versus the ability of the care team in the longer-term to treat the whole patient in a patient-centred and fully integrated manner.

**Kaiser:**

There are several types of integrated information systems architectures. One of these is the one deployed by Kaiser Permanente in the US. Kaiser’s information systems architecture contains the following elements: (a) a fully integrated electronic patient record at its core; (b) operation across care settings; (c) patients’ electronic access to their doctor and health record; (d) population care with whole patient chronic care management (for diabetes, COPD, congestive heart failure, asthma, etc.) with a consolidated disease register; (e) development and real-time deployment of embedded clinical protocols; (f) secure access by remote health facilities; (g) centralised technical standards and architecture alongside local developments (“think globally, act locally”); and (i) analytic tools for high volume, complex data.

**Integration:**

Integration architectures range from full functional integration to data interoperability. In full functional integration architectures, the electronic patient record is at the core. This patient record is the detailed (not summary) record and reflects a complex information system supporting the entire clinical process including: review of clinical data (results, images, documents), assessments, documentation and correspondence, requesting tests, prescribing and administering drugs, clinical decision support with real-time alerts, multi-resource scheduling, care plans and integrated care pathways, research and patient access to his/her record.

The fully integrated healthcare systems architecture applies to, and operates across, patients, clinicians, clinical teams, carers, social workers, GPs, community units and hospitals within the geographical community in which the patient lives and receives care.

**Conclusion:**

The recommended actions for UK telehealth and telecare projects are (a) define your systems architecture and its integration road map; (b) deploy road map and revise systems architecture; and (c) repeat to continuously improve information systems support for integrated care.

